# Intelligent ADL Recognition via IoT-Based Multimodal Deep Learning Framework

**DOI:** 10.3390/s23187927

**Published:** 2023-09-16

**Authors:** Madiha Javeed, Naif Al Mudawi, Abdulwahab Alazeb, Sultan Almakdi, Saud S. Alotaibi, Samia Allaoua Chelloug, Ahmad Jalal

**Affiliations:** 1 Department of Computer Science, Air University, E-9, Islamabad 44000, Pakistan; 191880@students.au.edu.pk; 2 Department of Computer Science, College of Computer Science and Information System, Najran University, Najran 55461, Saudi Arabia; naalmudawi@nu.edu.sa (N.A.M.); afalazeb@nu.edu.sa (A.A.); saalmakdi@nu.edu.sa (S.A.); 3 Information Systems Department, Umm Al-Qura University, Makkah 24382, Saudi Arabia; ssotaibi@uqu.edu.sa; 4 Department of Information Technology, College of Computer and Information Sciences, Princess Nourah bint Abdulrahman University, Riyadh 11671, Saudi Arabia

**Keywords:** activities of daily living recognition, deep learning, IoT, multimodal data, patient monitoring, smart homes

## Abstract

Smart home monitoring systems via internet of things (IoT) are required for taking care of elders at home. They provide the flexibility of monitoring elders remotely for their families and caregivers. Activities of daily living are an efficient way to effectively monitor elderly people at home and patients at caregiving facilities. The monitoring of such actions depends largely on IoT-based devices, either wireless or installed at different places. This paper proposes an effective and robust layered architecture using multisensory devices to recognize the activities of daily living from anywhere. Multimodality refers to the sensory devices of multiple types working together to achieve the objective of remote monitoring. Therefore, the proposed multimodal-based approach includes IoT devices, such as wearable inertial sensors and videos recorded during daily routines, fused together. The data from these multi-sensors have to be processed through a pre-processing layer through different stages, such as data filtration, segmentation, landmark detection, and 2D stick model. In next layer called the features processing, we have extracted, fused, and optimized different features from multimodal sensors. The final layer, called classification, has been utilized to recognize the activities of daily living via a deep learning technique known as convolutional neural network. It is observed from the proposed IoT-based multimodal layered system’s results that an acceptable mean accuracy rate of 84.14% has been achieved.

## 1. Introduction

Smart homes-based monitoring via IoT devices is an important concept to be taken into consideration [1,2]. Elderly and patient monitoring at IoT-based smart homes or facilities is a big challenge in this era [3]. Machines are not intelligent enough to take care of such patients at facilities by themselves [4]. Therefore, continuous improvements are needed when it comes to dealing with human health monitoring [5,6]. However, the standard approaches are less efficient and require a multimodal IoT-based methodology to provide robust monitoring systems [7,8]. Activities of daily living (ADLs) need to be examined for smart home monitoring systems. ADL monitoring applications are widespread including fall detection, home surveillance, smart environments, assistive robotics, and ambient assisted living [9,10,11,12,13,14]. ADLs are difficult to be recognized as each ADL consists of multiple small actions performed together to make one long activity [15]. Single type of raw sensor data are not able to detect the complex sequences of ADL. Different subjects can perform a single ADL by performing the small actions in a diverse sequence of actions [16]. Therefore, a robust multimodal IoT-based intelligent system is required to take care of these limitations [17].

Deep learning models can help machines to infer the natural intuitions of human body motion. They provide a great opportunity to learn through sufficient examples of human actions in ADL in order to then identify them [18]. End-to-end deep learning techniques are effective for high-level features extraction [19]. A deep learning framework will help facilities to cope with high costs and nursing shortages via ADL recognition [20]. Multiple hyper parameters can be used for each deep learning model to adjust the ADL recognition [21]. Therefore, we have proposed a unique framework for the ADL recognition of elderly people at smart homes and facilities using IoT-based multisensory devices. This study has suggested a systematic method to take multimodal data from many IoT devices and process them to remove any noise and bias. Next, human silhouette detection and features processing is performed to highlight the important characteristics of the system. Finally, these features are optimized and the ADL is classified using a deep learning model.

Two publicly available datasets based on multimodal sensors and videos have been used to perform the evaluation for our proposed method, namely, Opportunity++ [22] and Berkeley-MHAD [23]. These datasets contain numerous types of data, including inertial and vision-based data. The key contributions of this research paper are:

- A novel algorithm has been proposed for 2D stick model extraction in this study for supporting more efficient ADL recognition in less computational time.
- An algorithm for human body landmarks detection has been proposed to effectively recognize the daily locomotion activities.
- A genetic algorithm has been optimized using a state-of-the-art fitness formula proposed for video and inertial sensors-based ADL data.
- The proposed layers of the ADL recognition model support the delivery of a robust IoT-based multimodal system to achieve extraordinary efficiency.

A literature review is presented in Section 2 and a detailed architecture argument about the proposed IoT-based multimodal system is provided in Section 3. The experiments performed are described in Section 4 and this study’s conclusive remarks along with some future directions are offered in Section 5.

## 2. Literature Review

This section presents a detailed literature review of both simple and multimodal IoT-based approaches for ADL recognition in smart environments. We have distributed the literature review into two sections, namely, simple modal systems and IoT-based multimodal systems.

### 2.1. Simple Modal Systems

In the literature, many researchers have worked to recognize ADL through different methodologies. A module encompassing different sensors-based fusion and features extraction has been proposed in [24]. Accelerometers, magnetometers, and gyroscopes have been used in different combinations for ADL recognition. This study is more focused on environment identification, which leads to a low performance in ADL recognition. The authors of [25] have proposed an IoT-based model for the remote health monitoring of patients. Different health sensors, such as pulse, temperature, and galvanic skin response sensors were used. However, the system lacked actual implementation and could not perform well in the real-time environment. In [26], M. Sridharan et al. have proposed a model to map the location of activities performed by using already-detected landmarks and zones inside the home. They have also detected the gait of a person in different zones of the home. The model achieved 85% accuracy for trajectory prediction. However, due to no processing in the layers of filtration and features, the system attained a good performance with low-level information for ADL recognition.

A methodology consisting of four stages has been suggested in [27]. The four stages include acquisition, processing, fusion, and classification and have been described in the paper. The classification stage contained recognition of ADL, the identification of the environment, and the detection of activities with no motion involved. However, the lower the number of sensors utilized for classification, the less accurate the proposed methodology was. The researchers in [28] have proposed a study presenting an activity classification system analyzed over light gradient boosting, gradient boosting, cat boosting, extreme gradient boosting, and AdaBoost classifiers. A smartphone-based dataset has been utilized to test the performance and a few limitations were also present in the study, as in the ADL performance context.

In [29], an ADL recognition module has been proposed using video cameras. First, the data from cameras are acquired and pre-processed. Next, objects and humans along with their interactions are detected via two neural networks. Then, the activities are recognized through another neural network. Finally, the data are post-processed and transmitted to the gateway using priority queues, where a smartcare system has been introduced to use the results and monitor patients. However, a single sensor like camera-based activity recognition system is not a robust system. The authors explained inertial sensor-based ambient assisted living in [30]. They have denoised the signal using Chebyshev, Kalman, and dynamic data reconciliation filters. Next, windows of seven seconds each have been extracted from the signal. Then, signals are normalized and signal energy, variance, frequency, and empirical mode decomposition features are mined. Furthermore, the features are dimensionally reduced using Isomap and the activities are classified using CNN-biLSTM classifiers. However, while simple activities are recognized in the proposed method, it is not a robust approach towards complex ADLs present in the daily routine.

### 2.2. IoT-Based Multimodal Systems

Different multimodal systems have been proposed in approaches proposed by researchers. An audio and depth modalities-based ADL recognition system has been proposed in [31]. CNN has been used to recognize ADLs from depth videos, alhough the system was not applicable to real-time ADL recognition due to its computationally expensive nature. In [32], an ADL recognition approach using two deep learning methods has been suggested. The input has been provided to both CNN and bidirectional long short-term memory, and CNN layers performed direct mapping. However, using a grid search method to tune the hyper parameters has been very computationally expensive and thus this approach is not a feasible solution for real-time ADL recognition.

Due to differences in age, gender, weight, height etc., the authors proposed personalized models in [33]. Personalization makes it possible for machine learning algorithms to objectively evaluate the performance of proposed systems. It also considered the resemblances between the physical and signal forms. However, the accuracy improvements for physical, signal, and both fused together are not very impressive. Another hybrid approach using both motion sensors and cameras has been suggested in [34]. A motion–state layer and an activity layer have been used along with long-short-term-memory and CNN to recognize ADLs. Motion sensor data improved the classification according to the motion state while videos are utilized for the specification of ADL. However, due to the grouping of the motion state layer, the system was not able to produce acceptable results.

In [35], Žarić et al. presented a system to monitor the cooking process in home kitchens and to identify critical conditions related to elderly people. The proposed system utilized humidity, ultrasound, and temperature sensors as input to a system that is capable of generating an alert or a warning in case of a dangerous situation. They have also identified some cases for the analysis of the cooking process. A Moore finite-state machine having different states to the activities performed has been used to generate outputs using the proposed decision-making system. Nevertheless, the proposed system is limited to the kitchen environment and it is designed and tested only for electrical cooking plates. The authors of [36] described an ADL recognition and fall detection system using an Mbient sleeve sensor research kit, Imou smart cameras, proximity sensors, and the Microsoft SQL Server. They have given four concepts for fall detection including pose detection, data collection and processing, learning, and performance measurement. The complex activities have been further divided into atomic actions to detect the indoor localization. Then, the semantic relationship is inferred, studied, analyzed, and interpreted between accelerometer, gyroscope, and associated actions. Further, the integrated data are split into training and testing sets and accuracy has been computed. However, the system could achieve an accuracy of 81.13% due to the real-time environment and associated costs. The system focused on limited activities performed by the subjects whereas its performance is not clear when it comes to several other ADLs.

## 3. Materials and Methods

**Figure 1:**
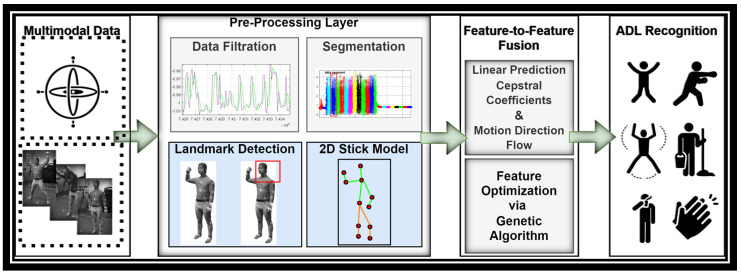
The architecture diagram for multimodal IoT-based deep learning framework via ADL recognition.

This system consists of two types of data, inertial and videos. A multimodality-based system has been proposed to recognize the complex forms of ADLs. It also aids recognition of ADLs where there are some data missing from one sensor. The inertial data have been filtered using Butterworth and the video frame sequences have been filtered by subtracting background from the frames. Furthermore, the landmarks have been detected from the filtered frame sequences and the filtered inertial data have been divided into windows of 5 s each. Then, the pre-processed data have been given to the features engineering layer to extract and reduce the huge number of features. Lastly, an ADL recognition layer has been utilized to classify the ADL from both state-of-the-art datasets. A detailed architecture diagram for a multimodal IoT-based deep learning framework is shown in Figure 1. The following subsections further explain each layer of this architecture for ADL recognition.

### 3.1. Pre-Processing of Inertial Sensor Signals

**Figure 2:**
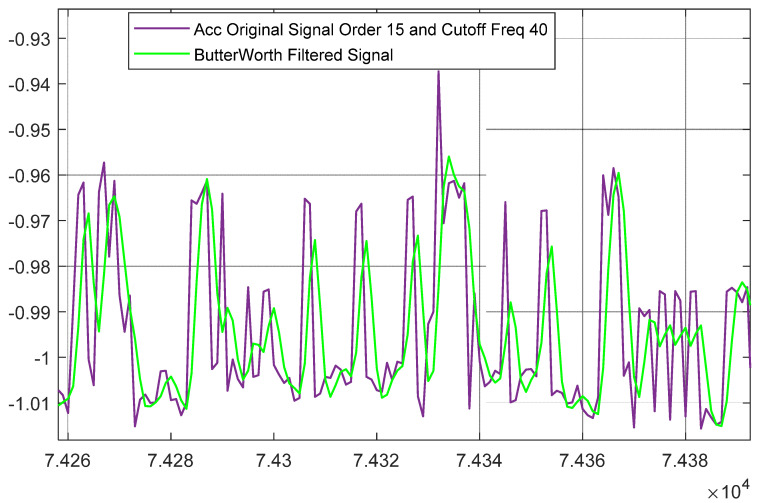
Sample signals after filters applied for motion sensor data.

Three different types of data have been retrieved from the inertial measurement unit, such as accelerometer, gyroscope, and magnetometer data. The acceleration data for ADL have been provided through accelerometer sensors. The gyroscope measures the angular velocity or the rate of change in sensors’ orientation. Magnetometers give a point of reference for measuring the strength and direction of magnetic fields, which is important in order to obtain a precise locomotion. There is noise present in all types of raw data attained from the sensors including the inertial data. Subsequently, to remove this noise, this study proposes a filter utilization to get an as low as possible response frequency known as the Butterworth filter [37]. Figure 2 shows the acceleration signal before and after applying the Butterworth filter to inertial data.

**Figure 3:**
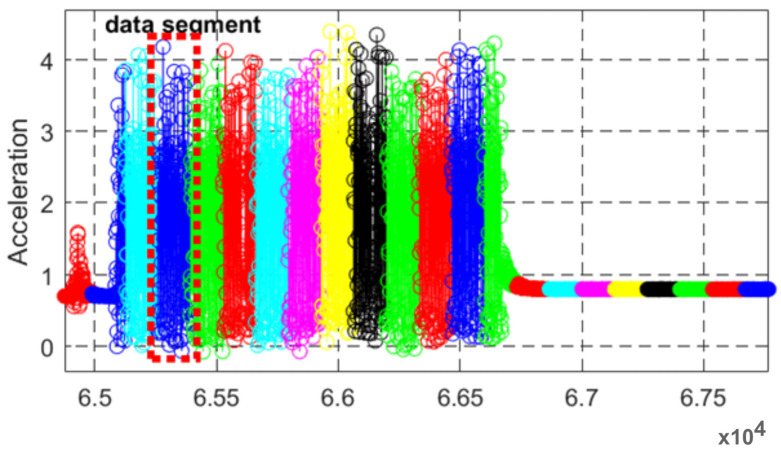
Detailed view of data segmentation applied over the inertial signal has been presented using multiple colors in the figure. The red dotted box shows single segment of data.

In preprocessing layer for inertial data and to help process in next layer this filtered data properly without any missing values, we proposed to utilize the data segmentation technique. After the filtration of raw data, the inertial signals have been segmented using an overlapping windowing procedure [38]. Figure 3 gives a detailed view of data segmentation applied over acceleration signal. Each color in the figure represents a data segment from the signal.

### 3.2. Pre-Processing of Videos

**Figure 4:**
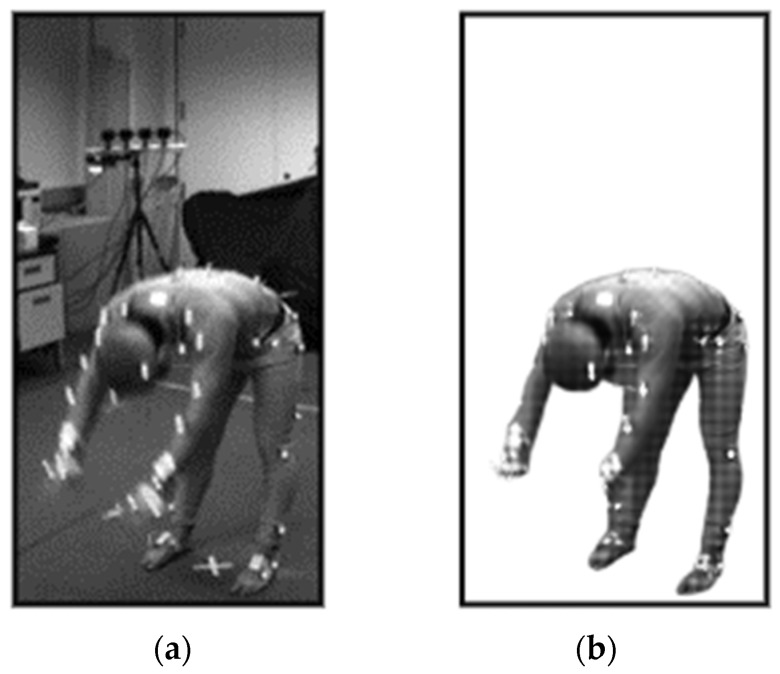
(**a**) Real video frame and (**b**) extracted human figure after background extraction for bending activity in Berkeley-MHAD dataset.

To produce accurate results, there is a need to process the input videos. First, the frames have been converted and the extracted images have been resized [39]. Then, the background has been subtracted from the frame sequences in order to get human silhouette for further processing. Figure 4 displays the human silhouette extracted after background subtraction. Afterwards, the head landmark has been detected using the human body shape and size [40] and the lowest point of body has been taken as the foot point of the human, calculated as:

(1) $$T_{Fo}^{f}\leftarrow \;T_{Fo}^{f-1}+{∆T}_{Fo}^{f-1},$$

where $T_{Fo}^{f}$ signifies the foot landmark position in the f frame sequences calculated using the frames variance. The calculations for human position has been designed as:

(2) $$T_{HS}^{f}=\left(T_{Fo}^{f}\leftarrow T_{Fo}^{f-1}+{∆T}_{Fo}^{f-1}\right)+T_{E}^{f},$$

where $T_{HS}^{f}$ provides the human position in a frame f and $T_{E}^{f}$ denotes the boundary for the frame. From both the head and foot point, the midpoint torso has been extracted followed by the neck, knee, hip, elbow, and shoulder points.

**Figure 5:**
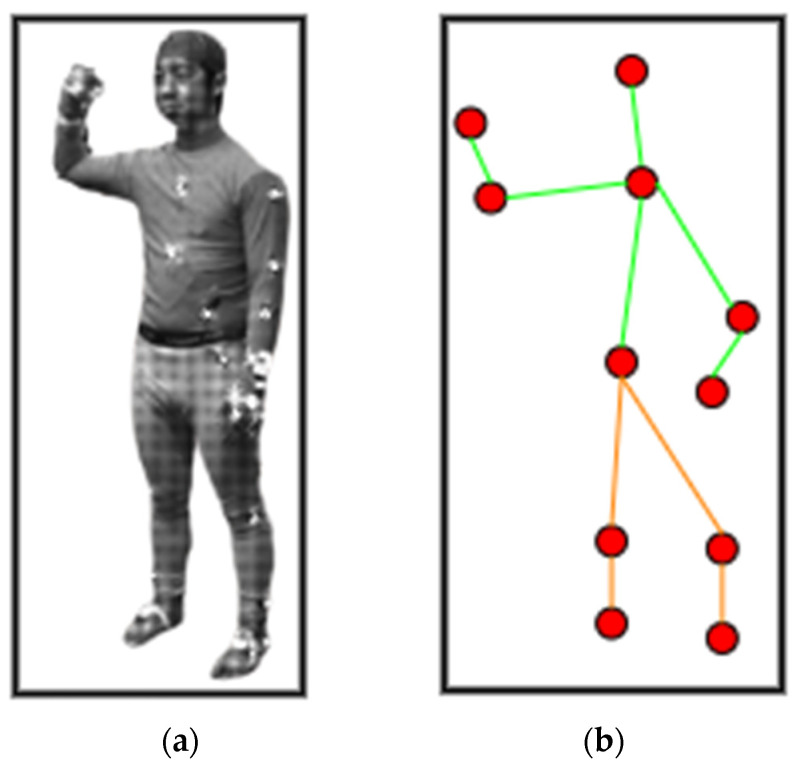
(**a**) Human silhouette (**b**) 2D stick model, where each red dot represents the body point detected, green lines show the upper body skeleton, and orange lines give the lower body skeleton.

After landmark detection, a 2D stick model [41] has been extracted through joining skeleton points detected from the mined landmarks as shown in Figure 5. Algorithm 1 describes the pre-processing layer in detail for landmark detection and 2D stick model development. First, the algorithm detects the head position and foot position in the human silhouette to be recognized as the landmarks. If the head position is detected, then other body landmarks are recognized and the mid-point of the recognized landmark is also detected. Next, the algorithm continues to detect the mid-points for each landmark detected. Lastly, when all the seven landmarks are detected, the stick model is extracted through connecting the mid-points.

**Algorithm 1:** Landmark detection and 2D stick model creation

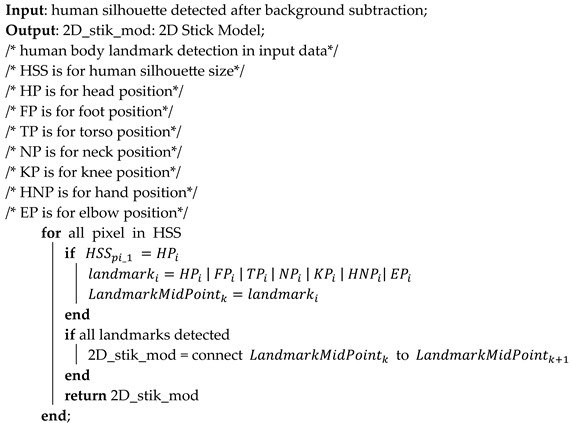



### 3.3. Features Processing Layer

**Figure 6:**
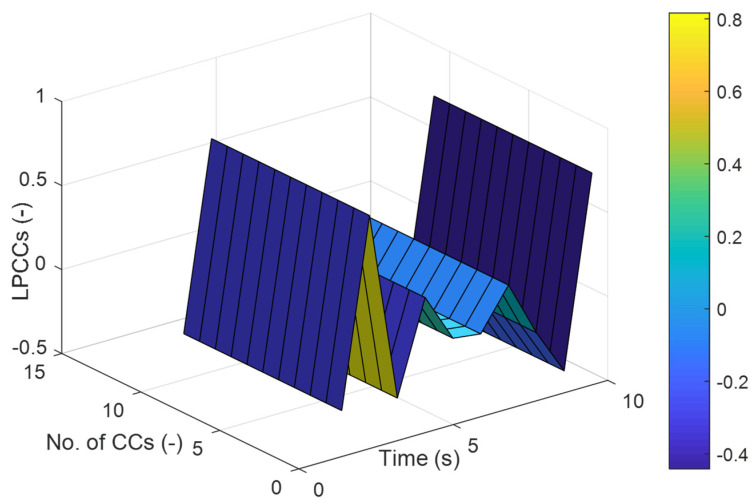
Extracted LPCCs result for the Jumping Jacks ADL over Berkeley-MHAD dataset.

In the second layer, we proposed to apply features extraction methodologies for both inertial and video data. Linear prediction cepstral coefficients (LPCCs) [42] have been applied for the inertial data using the equations:

(3) $${LPCC}_{o}=ln{.\sigma }^{2},$$

(4) $$lpcc_{m}=x_{m}+\sum_{n=1}^{m-1}\left(\frac{n}{m}\right)\;{lpcc}_{n}\;x_{m-n},\;1\leq m\leq p,$$

(5) $$lpcc_{m}=\sum_{n=1}^{m-1}\left(\frac{n}{m}\right)\;{lpcc}_{n}\;x_{m-n},\;p\;\leq m\leq e,$$

where $\sigma^{2}$ displays an estimate increase, $lpcc_{n}$ and $x_{m}$ denotes the LPCCs, and e conveys the LPCCs statistics. Figure 6 explicates the LPCCs extracted over jumping jacks activity over the Berkeley-MHAD dataset.

**Figure 7:**
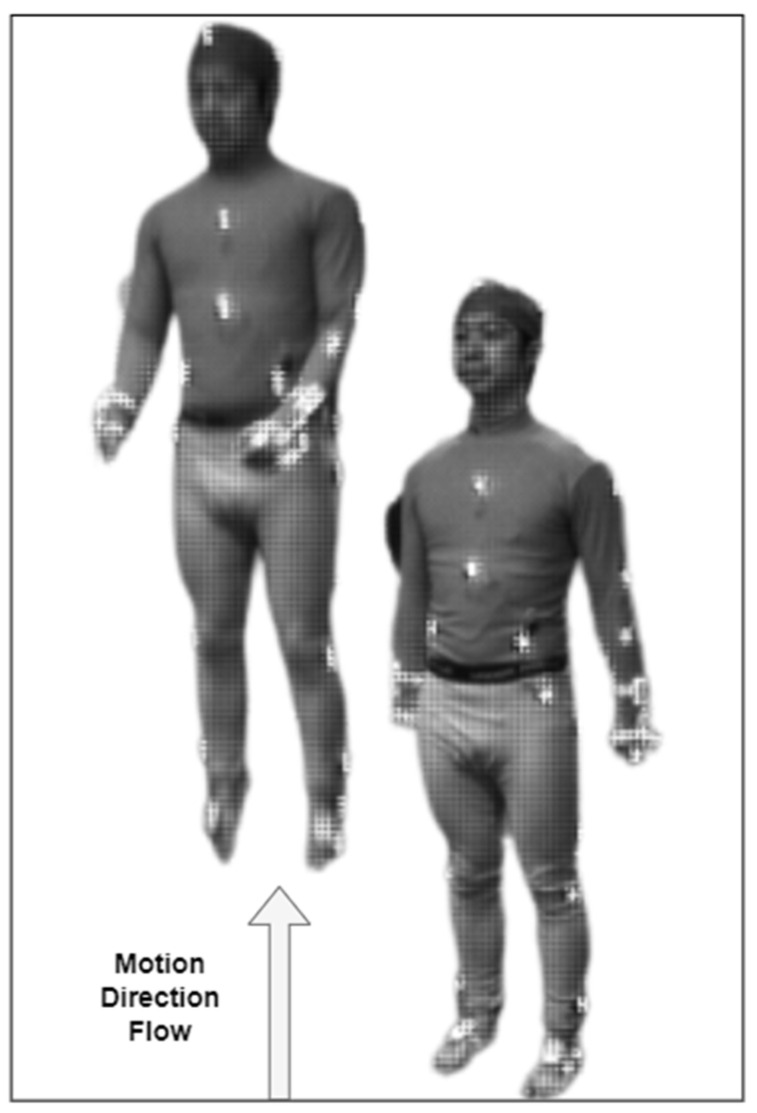
Upward motion direction flow in Jumping in Place ADL.

When it comes to predicting the ADL, the motion direction flow can significantly support the recognition of activities. It is a context-based feature that will identify the human movement patterns and directions [43]. The motion flow for the human body can be calculated as:

(6) $${Md}_{f}=\sum_{0}^{f}F_{v}\left(F\right)\to Md,$$

where F denotes the frame sequence extracted from video v, ${Md}_{f}$ gives the motion flow direction of the current frame sequence, and Md elucidates the motion flow direction from the previous frame. Figure 7 describes the motion direction flow for the jumping in place activity over the Berkeley-MHAD dataset.

**Figure 8:**
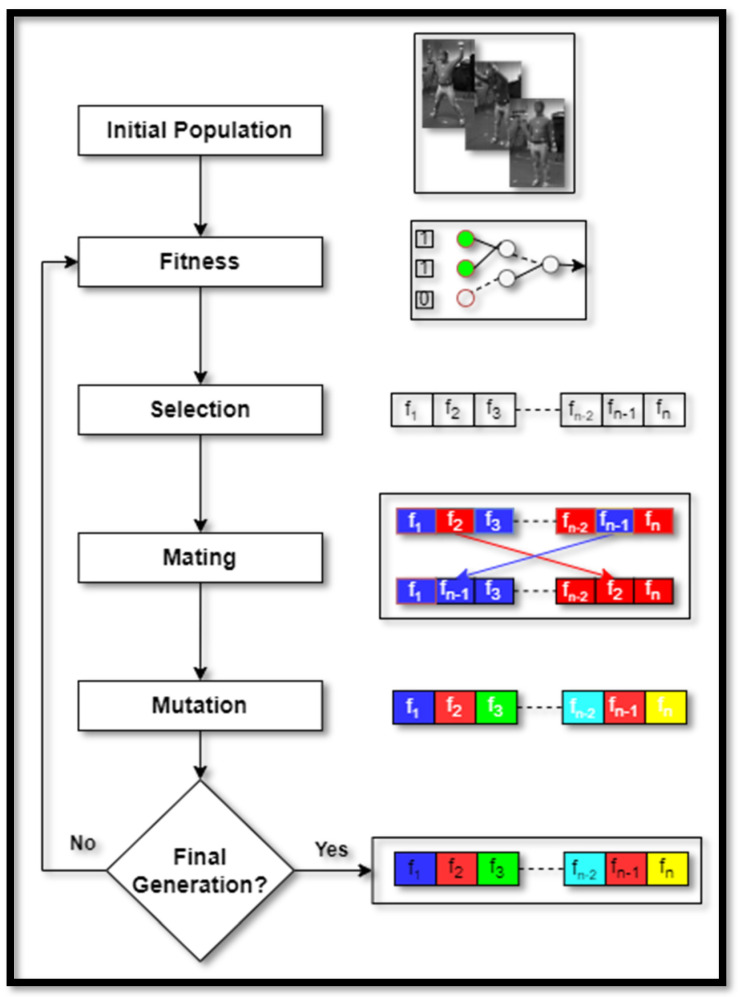
Features optimization via genetic algorithm explained through a detailed view.

After the features extraction stage, the dimensions of the feature vector have been increased immensely. Therefore, to reduce the feature vector size, we have introduced the application of the genetic algorithm [44]. It involves a few biological orders-based techniques including mutation, selection, mating, and crossover of the chromosomes. So, we have utilized the fitness formula mentioned as:

(7) $$fitness=x_{i}y_{i}+x_{f}y_{f}+\frac{\alpha }{f_{n}},$$

where $x_{i}$ denotes the scaling factor selected for inertial-based features, $y_{i}$ gives the average for all subjects in both datasets for inertial-based features, $x_{f}$ provides the scaling factor chosen for frame sequences-based features, $y_{f}$ shows the average over all subjects in both datasets for frame sequences-based features, $f_{n}$ denotes the number of features representing chromosomes, and α determines the scale factor set to 0.5. The detailed view of the genetic algorithm is represented in Figure 8.

### 3.4. ADL Recognition Layer

**Figure 9:**
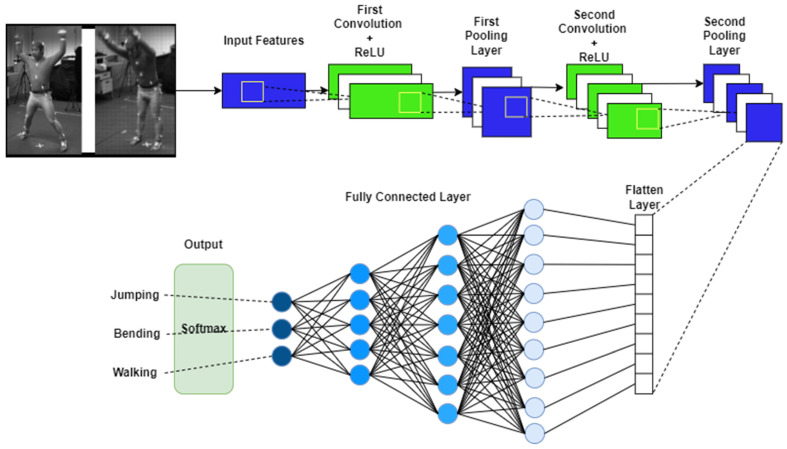
Proposed CNN model for multimodal IoT-based ADL recognition over Berkeley-MHAD.

CNN [45] takes both data types and gives weights along with bias to different features and classifies one activity from another. It is considered to be the most effective algorithm for recognition, retrieval, and classification. Multiple layers-based variants are being used by the researchers in the literature. It also contains three types of layers, such as input, hidden, and output layers. Each hidden layer contains multiple combinations of softmax, convolution, completely connected, and pooling layers. It also consists of activation functions used for the setting of each node, which was selected as a rectified linear unit (ReLU) [46]. We set the learning rate to 0.002 and the maximum epoch number was selected as 100. Figure 9 helps in understanding the CNN model for the ADL recognition layer. The input layer consisted of an activation shape in the form of (32, 32, 3) with an activation size of 3072 and no parameters. Next, the first convolution layer consisted of a (28, 28, 8) activation shape of ReLU along with a 6272 activation size, and 608 parameters with 5 filters. Then, the first pooling layer was utilized containing a (14, 14, 8) activation shape and 1568 size with 0 parameters. Further, the second convolutional layer has been added with a (10, 10, 16) activation shape and 1600 size along with 5 filters and 3216 parameters. Moreover, a second pooling layer consisted of a (5, 5, 16) activation shape and 400 size with 0 parameters. A flattened layer was further used. Two fully connected layers with (120, 1) and (84, 1) activation shapes and 120 and 84 size with 48,120 and 10,164 parameters were introduced next. Finally, a softmax layer of (10, 1) shape and 10 size in activation with 850 parameters has been used.

## 4. Dataset Experimental Setup and Results

A brief overview of the datasets utilized, experiments performed on them, and their results is discussed in this section.

### 4.1. Datasets Description: Berkeley-MHAD and Opportunity++

**Figure 10:**
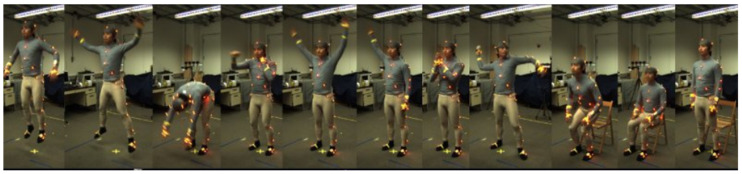
Sample frame sequences from the Berkeley-MHAD [22] dataset.

**Figure 11:**
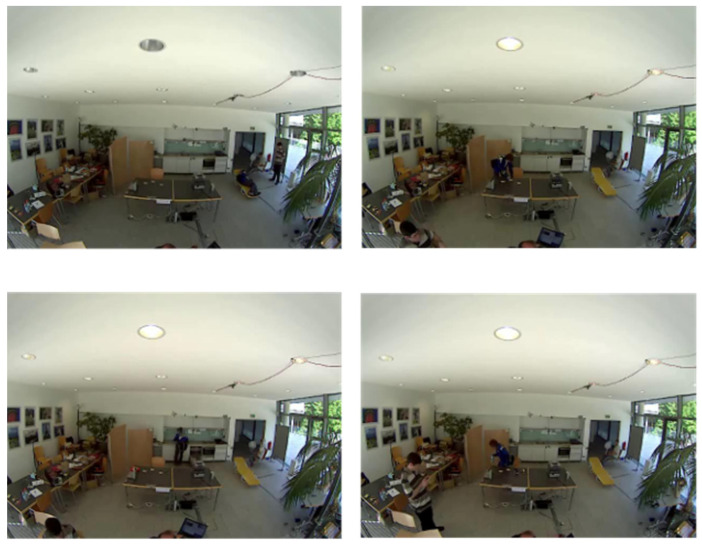
Sample frame sequences from Opportunity++ [21] dataset.

An open access, and one of the earliest multimodal datasets, named Berkeley-MHAD [23] has been used in this system to validate the experimental section. It contains 12 IoT-based ADLs performed in an indoor environmental setting. Figure 10 presents the sample frame sequences from the Berkeley-MHAD dataset. Another publicly available dataset called Opportunity++ [22] is utilized to perform experiments on the proposed ADL model. A total of 12 subjects performed different IoT-based ADLs, completed in an indoor environment. Figure 11 shows the sample frame sequences from the Opportunity++ dataset. In order to obtain a less-biased and less-optimistic estimate of the proposed ADL recognition system, we have used a 10 fold cross-validation technique to evaluate the system’s accuracy. The datasets have been shuffled randomly and split into 10 groups. For each group, it is tested and remaining groups are used to train the proposed ADL recognition model. The evaluation score is extracted from each set of test groups and the model’s performance has been determined.

### 4.2. Experimental Settings and Results

All the calculations and experimentation has been performed on a DELL laptop with Intel^®^ Core™ i7 4th generation CPU @ 2.4 GHz and 64-bit windows 10 bought from Islamabad, Pakistan. The software used was MATLAB (R2017a) for complete experimentation along with a 24 GB RAM.

#### 4.2.1. Experiment 1: Confusion Matrices over Opportunity++ and Berkeley-MHAD

This subsection describes the confusion matrices extracted for the ADL recognition experiments performed on the Berkeley-MHAD and Opportunity++ datasets. Table 1 and Table 2 provide a detailed explanation of true positives, false positives, true negatives, and false negatives [47,48,49] attained over both datasets with the recognition through CNN.

#### 4.2.2. Experiment 2: Confidence Levels over Skeleton Points

We also calculated the confidence levels detected for each part of the body identified in the landmark detection and 2D stick model generation stages. Table 3 gives a detailed view of 11 body points identified along with their confidence levels [50,51,52] in the range [0, 1]. The mean accuracies of 84.12% and 84.17% have been achieved by the proposed IoT-based multimodal system over Opportunity++ and Berkeley-MHAD datasets, respectively.

#### 4.2.3. Experiment 3: Comparison with Other Important Classifiers

In this section, we have further assessed the proposed system based on a comparison with two well-known classification methods—artificial neural network (ANN) [53,54] and AdaBoost [55,56] classifiers. Both models were trained using the scikit-learn library. For ANN, we used an input layer, two hidden layers, and an output layer. Each hidden layer contains 50 neurons and gradient descent with momentum has been selected as the learning algorithm. The minimum batch size is 50, momentum is 0.15, number of epochs is 500, and biases were initialized with 0. Initial weights are selected randomly from a normal distribution and learning decay is exponential. For Adaboost, we have set the base learners as decision tree with a maximum depth of 5 levels and the number of base estimators as 50. Learning rate has been set to 0.001 to avoid unnecessary delays during the testing phase and estimator weights have been chosen randomly.

It is evident from the Table 4 and Table 5 that our proposed model has achieved higher precision, recall [57], and *F*1-score [58] in both selected datasets, which shows that the multimodal IoT-based ADL recognition system using CNN has outperformed the others. The following are the equations for precision, recall, and *F*1-score:

(8) p=TP/(TP+FP),

(9) r=TP/(TP+FN),

(10) F−m=(2∗(r∗p))/(r+p),

where p is the precision, r is the recall, and F−m is the *F*1-score. True positives are determined from TP, false positives are given by FP, false negatives are displayed by FN, and true negatives are shown by TN.

#### 4.2.4. Experiment 4: Comparison with Other State-Of-The-Art Techniques in Literature

Further, to validate the performance of the proposed IoT-based recognition system, we have given a comparison in Table 6 with other state-of-the-art methodologies presented in the literature. It is evident from the table that our proposed system outperformed the others in terms of accuracy for Opportunity++ [59,60] and Berkeley-MHAD datasets [61,62,63].

## 5. Discussion

The proposed ADL recognition system has focused on the usage of IoT-based devices for collecting data from humans, including elderly people and patients at a certain place. The data collected can be in the form of videos, their sequences, audio, and locks etc. A smart home or a private room in a hospital is a person’s private and protected space. These IoT-based devices give rise to privacy and protection concerns, which can be mitigated by introducing multiple privacy mechanisms. Some studies proposed to introduce a minimum ratio of noise into the data in order to protect the privacy of a home [64,65,66,67]. A few articles proposed to provide an infrastructure for such devices that can send personalized notices and give the choice to obtain a person’s user preferences [68,69,70]. Overall, an auto configuration support system has also been proposed in order to make sure that whenever a new device has been attached to the existing system, it is auto-configured according to the security protocols and user preferences [71,72,73]. However, in the selected datasets for the proposed article, the faces of the individuals have also been blurred to maintain the privacy of users [74,75,76].

**Figure 12:**
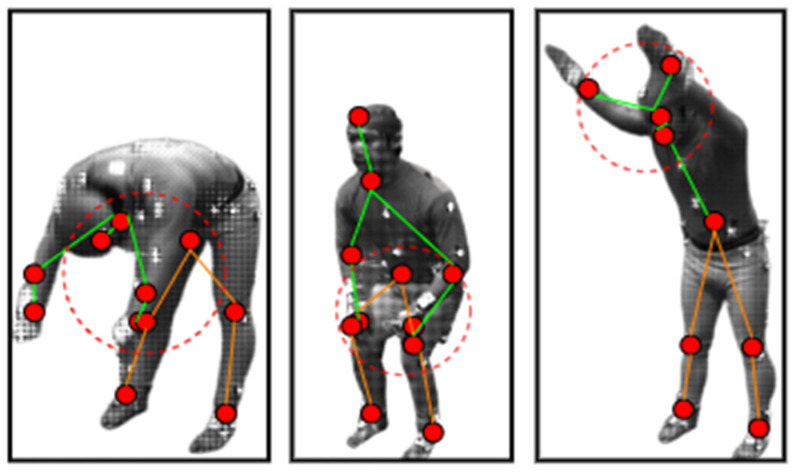
Examples of problematic ADL activities over Berkeley-MHAD, where red dotted circles point out the skeleton extraction problems.

ADL recognition has been achieved successfully using the proposed model with landmark detection and a 2D stick model along with inertial sensor signal processing. We had to extract different body points in this method to make the 2D stick model. However, there were few ADL that could not achieve the ideal 2D stick model shape and caused the accuracy rates to decrease. Figure 12 gives examples of such activities performed during the ADL recognition stage. The landmark areas pointed out by red dotted circles show that the body landmarks’ mid-points can be mixed up in specific body postures, therefore causing the performance of the 2D stick model and the accuracy rate to be compromised.

## 6. Conclusions and Future Work

Our proposed method for IoT-based ADL recognition is an important novel idea for the elderly home monitoring system. It is a combination of multimodal-based sensors to compute the ADL recognition efficiently. First, the multimodal data are filtered through multiple types of filtering techniques. Next, the inertial data are segmented using windows and vision data have been used to find the landmarks and create the 2D stick model. Then, we used state-of-the-art techniques like LPCCs and motion direction flow determination for inertial and video data, respectively. Further, to reduce the dimensionality issue, we proposed to utilize the genetic algorithm with a novel fitness function. Lastly, an efficient deep learner known as CNN has been applied over the reduced features to classify the ADL. Mean accuracies of 84.12% and 84.17% have been achieved over Opportunity++ and Berkeley-MHAD datasets. The results have shown that the proposed ADL recognition technique has outperformed in certain ways, such as confidence levels of body landmarks detection, accuracy rate of the system, and other state-of-the-art methodologies-based comparisons.

In the future we will focus on the privacy issues and improvement of the 2D stick model. Another shortcoming worth-mentioning is that the proposed system removed background from the videos provided by immobile indoor cameras. However, this study might not work when there are different background settings in the data. Thus, the system will be implemented over more generalized environmental settings and data.

## Figures and Tables

**Table 1:** Confusion matrix for ADL recognition for proposed approach recognition through CNN over the Opportunity++.

IoT-Based ADL	OD1	OD2	CD1	CD2	OF	CF	ODW	CDW	ODW1	CDW1	ODW2	CDW2	ODW3	CDW3	CT	DC	TS
OD1 *	8	0	1	0	0	0	1	0	0	0	0	0	0	0	0	0	0
OD2	0	9	0	0	0	0	0	0	0	0	0	0	1	0	0	0	0
CD1	0	0	9	0	0	0	0	1	0	0	0	0	0	0	0	0	0
CD2	0	0	1	8	0	0	0	0	0	1	0	0	0	0	0	0	0
OF	1	0	0	0	9	0	0	0	0	0	0	0	0	0	0	0	0
CF	0	0	0	0	0	8	0	0	0	1	0	0	0	0	1	0	0
ODW	0	0	0	0	1	0	8	0	0	0	0	0	0	1	0	0	0
CDW	0	1	0	0	0	0	0	9	0	0	0	0	0	0	0	0	0
ODW1	0	0	0	0	0	0	0	0	8	0	0	1	0	0	0	1	0
CDW1	0	0	0	1	0	0	0	1	0	8	0	0	0	0	0	0	0
ODW2	0	0	0	0	0	0	0	1	0	0	9	0	0	0	0	0	0
CDW2	0	0	0	0	0	0	0	0	0	0	0	9	0	0	0	0	1
ODW3	2	0	0	0	0	0	0	0	0	0	0	0	8	0	0	0	0
CDW3	0	0	0	0	1	0	0	0	1	0	0	1	0	8	0	0	0
CT	0	0	0	0	0	0	0	0	0	0	0	1	0	0	9	0	0
DC	0	0	0	0	0	1	0	0	0	1	0	0	0	1	0	**8**	0
TS	0	0	0	0	0	0	0	0	0	0	0	0	1	0	0	1	**8**
Mean accuracy = 84.12%																	

* OD1 = Open Door 1, OD2 = Open Door 2, CD1 = Close Door 1, CD2 = Close Door 2, OF = Open Fridge, CF = Close Fridge, ODW = Open Dishwasher, CDW = Close Dishwasher, ODW1 = Open Drawer 1, CDW1 = Close Drawer 1, ODW2 = Open Drawer 2, CDW2 = Close Drawer 2, ODW3 = Open Drawer 3, CDW3 = Close Drawer 3, CT = Clean Table, DC = Drink from cup, TS = Toggle Switch.

**Table 2:** Confusion matrix for ADL recognition for proposed approach recognition through CNN over the Berkeley-MHAD.

IoT-Based ADL	JIP	JJ	Ben	Pun	WaT	WaO	CH	TB	SiT	SD	SU	TP
JIP *	9	0	0	0	0	1	0	0	0	0	0	0
JJ	0	8	0	0	1	0	0	0	0	1	0	0
Ben	1	0	9	0	0	0	0	0	0	0	0	0
Pun	0	0	1	8	0	0	0	0	1	0	0	0
WaT	0	0	0	0	9	0	0	0	0	0	0	1
WaO	0	1	0	0	0	8	1	0	0	0	0	0
CH	0	0	0	1	0	0	8	0	0	0	1	0
TB	1	0	0	0	0	1	0	8	0	0	0	0
SiT	0	0	0	0	1	0	0	0	9	0	0	0
SD	0	0	1	0	1	0	0	0	0	8	0	0
SU	0	0	0	0	0	0	0	0	0	0	9	1
TP	0	0	0	0	1	0	0	1	0	0	0	**8**
Mean accuracy = 84.17%												

* JIP = Jumping in place, JJ = Jumping jacks, Ben = Bending, Pun = Punching, WaT = Waving-Two hands, WaO = Waving-One hand, CH = Clapping hands, TB = Throwing a ball, SiT = Sit down then stand up, SD = Sit down, SU = Stand up, TP = T-pose.

**Table 3:** Confidence levels over Berkeley-MHAD and Opportunity++ for body points detected.

Human Skeleton Points	Confidence Level for Berkeley-MHAD	Confidence Level for Opportunity++
Head	0.83	0.85
Neck	0.99	0.98
Right Elbow	0.83	0.85
Left Elbow	0.81	0.88
Right Wrist	0.74	0.78
Left Wrist	0.77	0.78
Torso	0.87	0.88
Right knee	0.79	0.84
Left knee	0.65	0.75
Right ankle	0.67	0.66
Left ankle	0.71	0.77
Mean Confidence	0.72	0.75

**Table 4:** Comparative analysis with other well-known classifiers in terms of precision and recall over Berkeley-MHAD dataset.

Locomotor Activities	Artificial Neural Network			AdaBoost			CNN		
	Precision	Recall	F1-Score	Precision	Recall	F1-Score	Precision	Recall	F1-Score
JIP	0.78	0.77	0.77	0.80	0.81	0.80	0.90	0.82	0.85
JJ	0.74	0.71	0.72	0.73	0.78	0.75	0.80	0.89	0.84
Ben	0.77	0.74	0.75	0.77	0.78	0.77	0.90	0.82	0.85
Pun	0.70	0.72	0.70	0.73	0.71	0.71	0.80	0.89	0.84
WaT	0.77	0.79	0.77	0.81	0.82	0.81	0.90	0.69	0.78
WaO	0.81	0.80	0.80	0.88	0.87	0.87	0.80	0.80	0.80
CH	0.74	0.80	0.76	0.79	0.75	0.76	0.80	0.89	0.84
TB	0.77	0.77	0.77	0.71	0.75	0.72	0.80	0.89	0.84
SiT	0.79	0.88	0.83	0.85	0.86	0.85	0.90	0.90	0.90
SD	0.76	0.77	0.76	0.79	0.78	0.78	0.80	0.89	0.84
SU	0.81	0.82	0.81	0.74	0.76	0.74	0.90	0.90	0.90
TP	0.82	0.84	0.82	0.88	0.90	0.88	0.80	0.80	0.80
Mean	0.77	0.78	0.77	0.79	0.80	0.78	0.84	0.85	0.84

**Table 5:** Comparative analysis with other well-known classifiers in terms of precision and recall over Opportunity++ dataset.

Locomotor Activities	Artificial Neural Network			AdaBoost			CNN		
	Precision	Recall	F1-Score	Precision	Recall	F1-Score	Precision	Recall	F1-Score
OD1	0.82	0.87	0.84	0.77	0.79	0.77	0.80	0.73	0.76
OD2	0.74	0.71	0.72	0.80	0.73	0.76	0.90	0.90	0.90
CD1	0.77	0.79	0.77	0.78	0.80	0.78	0.90	0.82	0.85
CD2	0.73	0.75	0.73	0.77	0.71	0.73	0.80	0.89	0.84
OF	0.69	0.68	0.68	0.78	0.74	0.75	0.90	0.82	0.85
CF	0.85	0.81	0.82	0.74	0.85	0.79	0.80	0.89	0.84
ODW	0.64	0.68	0.65	0.61	0.63	0.61	0.80	0.89	0.84
CDW	0.87	0.81	0.83	0.77	0.76	0.76	0.90	0.75	0.81
ODW1	0.77	0.71	0.73	0.78	0.79	0.78	0.80	0.89	0.84
CDW1	0.72	0.73	0.72	0.80	0.79	0.79	0.80	0.73	0.76
ODW2	0.77	0.79	0.77	0.84	0.82	0.82	0.90	1.00	0.94
CDW2	0.83	0.81	0.81	0.80	0.80	0.80	0.90	0.75	0.81
ODW3	0.74	0.79	0.76	0.87	0.81	0.83	0.80	0.80	0.80
CDW3	0.89	0.88	0.88	0.78	0.80	0.78	0.80	0.80	0.80
CT	0.75	0.79	0.76	0.71	0.70	0.70	0.90	0.90	0.90
DC	0.88	0.89	0.88	0.80	0.86	0.82	0.80	0.80	0.80
TS	0.77	0.78	0.77	0.79	0.79	0.79	0.80	0.89	0.84
Mean	0.77	0.78	0.77	0.77	0.77	0.76	0.84	0.83	0.83

**Table 6:** Comparative analysis with other state-of-the-art techniques over both datasets.

State-Of-The-Art Systems	Opportunity++ Accuracy (%)	Berkeley-MHAD Accuracy (%)
PER System [59]	74.70	-
IoT-based System [60]	74.70	-
D-Mocap System [61]	-	84.00
3D Human Skeleton Model [62]	-	83.92
MHAD Multiview Motion capture Method [63]	-	84.00
Proposed ADL Recognition System	84.12	84.17

## Data Availability

Not applicable.

## References

[1] Ali M., Ali A.A., Taha A.-E., Dhaou I.B., Gia T.N. Intelligent Autonomous Elderly Patient Home Monitoring System. Proceedings of the ICC 2019—2019 IEEE International Conference on Communications (ICC).

[2] Madiha J., Ahmad J., Kim K. Wearable Sensors based Exertion Recognition using Statistical Features and Random Forest for Physical Healthcare Monitoring. Proceedings of the 2021 International Bhurban Conference on Applied Sciences and Technologies (IBCAST).

[3] Zhou X., Zhang L. (2022). SA-FPN: An effective feature pyramid network for crowded human detection. Appl. Intell..

[4] Liu Y., Wang K., Liu L., Lan H., Lin L. (2022). TCGL: Temporal Contrastive Graph for Self-Supervised Video Representation Learning. IEEE Trans. Image Process..

[5] Gaddam A., Mukhopadhyay S.C., Gupta G.S. Trial & experimentation of a smart home monitoring system for elderly. Proceedings of the 2011 IEEE International Instrumentation and Measurement Technology Conference.

[6] Zouba N., Bremond F., Thonnat M. An Activity Monitoring System for Real Elderly at Home: Validation Study. Proceedings of the 2010 7th IEEE International Conference on Advanced Video and Signal Based Surveillance.

[7] Chen J., Wang Q., Cheng H., Peng W., Xu W. (2022). A Review of Vision-Based Traffic Semantic Understanding in ITSs. IEEE Trans. Intell. Transp. Syst..

[8] Suryadevara N.K., Mukhopadhyay S.C., Rayudu R.K., Huang Y.M. Sensor data fusion to determine wellness of an elderly in intelligent home monitoring environment. Proceedings of the 2012 IEEE International Instrumentation and Measurement Technology Conference Proceedings.

[9] Madiha J., Gochoo M., Jalal A., Kim K. (2021). HF-SPHR: Hybrid Features for Sustainable Physical Healthcare Pattern Recognition Using Deep Belief Networks. Sustainability.

[10] Foroughi H., Aski B.S., Pourreza H. Intelligent video surveillance for monitoring fall detection of elderly in home environments. Proceedings of the 2008 11th International Conference on Computer and Information Technology.

[11] Bruno B., Mastrogiovanni F., Sgorbissa A. A public domain dataset for ADL recognition using wrist-placed accelerometers. Proceedings of the 23rd IEEE International Symposium on Robot and Human Interactive Communication.

[12] Nguyen T.-H.-C., Nebel J.-C., Florez-Revuelta F. (2016). Recognition of Activities of Daily Living with Egocentric Vision: A Review. Sensors.

[13] Gambi E., Temperini G., Galassi R., Senigagliesi L., De Santis A. (2020). ADL Recognition Through Machine Learning Algorithms on IoT Air Quality Sensor Dataset. IEEE Sens. J..

[14] Nisar M.A., Shirahama K., Li F., Huang X., Grzegorzek M. (2020). Rank Pooling Approach for Wearable Sensor-Based ADLs Recognition. Sensors.

[15] Wang F., Wang H., Zhou X., Fu R. (2022). A Driving Fatigue Feature Detection Method Based on Multifractal Theory. IEEE Sens. J..

[16] Nasution A.H., Emmanuel S. Intelligent Video Surveillance for Monitoring Elderly in Home Environments. Proceedings of the 2007 IEEE 9th Workshop on Multimedia Signal Processing.

[17] Zhang Z., Cui P., Zhu W. (2022). Deep Learning on Graphs: A Survey. IEEE Trans. Knowl. Data Eng..

[18] Wang A., Zhao S., Zheng C., Yang J., Chen G., Chang C.-Y. (2021). Activities of Daily Living Recognition with Binary Environment Sensors Using Deep Learning: A Comparative Study. IEEE Sens. J..

[19] Ghayvat H., Pandya S., Patel A. Deep Learning Model for Acoustics Signal Based Preventive Healthcare Monitoring and Activity of Daily Living. Proceedings of the 2nd International Conference on Data, Engineering and Applications (IDEA).

[20] Zerkouk M., Chikhaoui B. (2020). Spatio-Temporal Abnormal Behavior Prediction in Elderly Persons Using Deep Learning Models. Sensors.

[21] Ciliberto M., Rey V.F.F., Calatroni A., Lukowicz P., Roggen D. (2021). Opportunity++: A Multimodal Dataset for Video- and Wearable, Object and Ambient Sensors-Based Human Activity Recognition. Front. Comput. Sci..

[22] Ofli F., Chaudhry R., Kurillo G., Vidal R., Bajcsy R. Berkeley MHAD: A comprehensive Multimodal Human Action Database. Proceedings of the 2013 IEEE Workshop on Applications of Computer Vision (WACV).

[23] Pires I.M., Marques G., Garcia N.M., Pombo N., Flórez-Revuelta F., Spinsante S., Teixeira M.C., Zdravevski E. (2019). Recognition of Activities of Daily Living and Environments Using Acoustic Sensors Embedded on Mobile Devices. Electronics.

[24] Hamim M., Paul S., Hoque S.I., Rahman M.N., Baqee I.-A. IoT Based Remote Health Monitoring System for Patients and Elderly People. Proceedings of the 2019 International Conference on Robotics, Electrical and Signal Processing Techniques (ICREST).

[25] Sridharan M., Bigham J., Campbell P.M., Phillips C., Bodanese E. (2020). Inferring Micro-Activities Using Wearable Sensing for ADL Recognition of Home-Care Patients. IEEE J. Biomed. Health Inform..

[26] Ferreira J.M., Pires I.M., Marques G., García N.M., Zdravevski E., Lameski P., Flórez-Revuelta F., Spinsante S., Xu L. (2020). Activities of Daily Living and Environment Recognition Using Mobile Devices: A Comparative Study. Electronics.

[27] Rahman S., Irfan M., Raza M., Moyeezullah Ghori K., Yaqoob S., Awais M. (2020). Performance Analysis of Boosting Classifiers in Recognizing Activities of Daily Living. Int. J. Environ. Res. Public Health.

[28] Madhuranga D., Madhushan R., Siriwardane C., Gunasekera K. (2021). Real-time multimodal ADL recognition using convolution neural network. Vis. Comput..

[29] Achirei S.-D., Heghea M.-C., Lupu R.-G., Manta V.-I. (2022). Human Activity Recognition for Assisted Living Based on Scene Understanding. Appl. Sci..

[30] Ghadi Y.Y., Batool M., Gochoo M., Alsuhibany S.A., Al Shloul T., Jalal A., Park J. (2022). Improving the ambient intelligence living using deep learning classifier. Comput. Mater. Contin..

[31] Ihianle I.K., Nwajana A.O., Ebenuwa S.H., Otuka R.I., Owa K., Orisatoki M.O. (2020). A Deep Learning Approach for Human Activities Recognition from Multimodal Sensing Devices. IEEE Access.

[32] Ferrari A., Micucci D., Mobilio M., Napoletano P. (2020). On the Personalization of Classification Models for Human Activity Recognition. IEEE Access.

[33] Yu H., Pan G., Pan M., Li C., Jia W., Zhang L., Sun M. (2019). A Hierarchical Deep Fusion Framework for Egocentric Activity Recognition using a Wearable Hybrid Sensor System. Sensors.

[34] Madiha J., Mudawi N.A., Alabduallah B.I., Jalal A., Kim W. (2023). A Multimodal IoT-Based Locomotion Classification System Using Features Engineering and Recursive Neural Network. Sensors.

[35] Žarić N., Radonjić M., Pavlićević N., Paunović Žarić S. (2021). Design of a Kitchen-Monitoring and Decision-Making System to Support AAL Applications. Sensors.

[36] Thakur N., Han C.Y. (2022). A Simplistic and Cost-Effective Design for Real-World Development of an Ambient Assisted Living System for Fall Detection and Indoor Localization: Proof-of-Concept. Information.

[37] Al Shloul T., Javeed M., Gochoo M., Alsuhibany S.A., Ghadi Y.Y., Jalal A., Park J. (2023). Student’s health exercise recognition tool for E-learning education. Intell. Autom. Soft Comput..

[38] Zhang J., Tang Y., Wang H., Xu K. (2023). ASRO-DIO: Active Subspace Random Optimization Based Depth Inertial Odometry. IEEE Trans. Robot..

[39] Akhtar I., Ahmad J., Kim K. (2021). Adaptive Pose Estimation for Gait Event Detection Using Context-Aware Model and Hierarchical Optimization. J. Electr. Eng. Technol..

[40] Akhter I., Hafeez S. Human Body 3D Reconstruction and Gait Analysis via Features Mining Framework. Proceedings of the 2022 19th International Bhurban Conference on Applied Sciences and Technology (IBCAST).

[41] Madiha J., Ahmad J. Body-worn Hybrid-Sensors based Motion Patterns Detection via Bag-of-features and Fuzzy Logic Optimization. Proceedings of the 2021 International Conference on Innovative Computing (ICIC).

[42] Shen Y., Ding N., Zheng H.-T., Li Y., Yang M. (2021). Modeling Relation Paths for Knowledge Graph Completion. IEEE Trans. Knowl. Data Eng..

[43] Madiha J., Chelloug S.A. Automated gestures recognition in Exergaming. Proceedings of the 2022 International conference on Electrical Engineering and Sustainable Technologies (ICEEST).

[44] Ghadi Y.Y., Javeed M., Alarfaj M., Al Shloul T., Alsuhibany S.A., Jalal A., Kamal S., Kim D.-S. (2022). MS-DLD: Multi-sensors based daily locomotion detection via kinematic-static energy and body-specific HMMs. IEEE Access.

[45] Javeed M., Shorfuzzaman M., Alsufyani N., Chelloug S.A., Jalal A., Park J. (2022). Physical human locomotion prediction using manifold regularization. PeerJ Comput. Sci..

[46] Wei H., Jafari R., Kehtarnavaz N. (2020). Fusion of Video and Inertial Sensing for Deep Learning–Based Human Action Recognition. Sensors.

[47] Zou W., Sun Y., Zhou Y., Lu Q., Nie Y., Sun T., Peng L. (2022). Limited Sensing and Deep Data Mining: A New Exploration of Developing City-Wide Parking Guidance Systems. IEEE Intell. Transp. Syst. Mag..

[48] Gumaei A., Hassan M.M., Alelaiwi A., Alsalman H. (2019). A Hybrid Deep Learning Model for Human Activity Recognition Using Multimodal Body Sensing Data. IEEE Access.

[49] Taylor W., Shah S.A., Dashtipour K., Zahid A., Abbasi Q.H., Imran M.A. (2020). An Intelligent Non-Invasive Real-Time Human Activity Recognition System for Next-Generation Healthcare. Sensors.

[50] Cheng B., Wang M., Zhao S., Zhai Z., Zhu D., Chen J. (2017). Situation-Aware Dynamic Service Coordination in an IoT Environment. IEEE/ACM Trans. Netw..

[51] Zhong T., Wang W., Lu S., Dong X., Yang B. (2023). RMCHN: A Residual Modular Cascaded Heterogeneous Network for Noise Suppression in DAS-VSP Records. IEEE Geosci. Remote Sens. Lett..

[52] Cao K., Ding H., Wang B., Lv L., Tian J., Wei Q., Gong F. (2022). Enhancing Physical-Layer Security for IoT With Nonorthogonal Multiple Access Assisted Semi-Grant-Free Transmission. IEEE Internet Things J..

[53] Abiodun O.I., Jantan A., Omolara A.E., Dada K.V., Umar A.M., Linus O.U., Arshad H., Kazaure A.A., Gana U., Kiru M.U. (2019). Comprehensive Review of Artificial Neural Network Applications to Pattern Recognition. IEEE Access.

[54] Li D., Ge S.S., Lee T.H. (2021). Fixed-Time-Synchronized Consensus Control of Multiagent Systems. IEEE Trans. Control Netw. Syst..

[55] Wang F., Li Z., He F., Wang R., Yu W., Nie F. (2019). Feature Learning Viewpoint of Adaboost and a New Algorithm. IEEE Access.

[56] Randhawa K., Loo C.K., Seera M., Lim C.P., Nandi A.K. (2018). Credit Card Fraud Detection Using AdaBoost and Majority Voting. IEEE Access.

[57] Zheng Y., Lv X., Qian L., Liu X. (2022). An Optimal BP Neural Network Track Prediction Method Based on a GA&ndash; ACO Hybrid Algorithm. J. Mar. Sci. Eng..

[58] Liao Q., Chai H., Han H., Zhang X., Wang X., Xia W., Ding Y. (2022). An Integrated Multi-Task Model for Fake News Detection. IEEE Trans. Knowl. Data Eng..

[59] Akhter I., Javeed M., Jalal A. Deep Skeleton Modeling and Hybrid Hand-crafted Cues over Physical Exercises. Proceedings of the 2023 International Conference on Communication, Computing and Digital Systems (C-CODE).

[60] Azmat U., Jalal A., Javeed M. Multi-sensors Fused IoT-based Home Surveillance via Bag of Visual and Motion Features. Proceedings of the 2023 International Conference on Communication, Computing and Digital Systems (C-CODE).

[61] Lannan N., Zhou L., Fan G. (2022). Human Motion Enhancement via Tobit Kalman Filter-Assisted Autoencoder. IEEE Access.

[62] Tian Y., Li H., Cui H., Chen J. (2022). Construction motion data library: An integrated motion dataset for on-site activity recognition. Sci. Data.

[63] Lannan N., Zhou L., Fan G. A Multiview Depth-based Motion Capture Benchmark Dataset for Human Motion Denoising and Enhancement Research. Proceedings of the 2022 IEEE/CVF Conference on Computer Vision and Pattern Recognition Workshops (CVPRW).

[64] Zhang X., Huang D., Li H., Zhang Y., Xia Y., Liu J. (2023). Self-training maximum classifier discrepancy for EEG emotion recognition. CAAI Trans. Intell. Technol..

[65] Li L., Wu X., Kong M., Liu J., Zhang J. (2023). Quantitatively Interpreting Residents Happiness Prediction by Considering Factor–Factor Interactions. IEEE Trans. Comput. Soc. Syst..

[66] Dai X., Xiao Z., Jiang H., Alazab M., Lui J.C.S., Dustdar S., Liu J. (2023). Task Co-Offloading for D2D-Assisted Mobile Edge Computing in Industrial Internet of Things. IEEE Trans. Ind. Inform..

[67] Jiang H., Xiao Z., Li Z., Xu J., Zeng F., Wang D. (2022). An Energy-Efficient Framework for Internet of Things Underlaying Heterogeneous Small Cell Networks. IEEE Trans. Mob. Comput..

[68] Lv Z., Qiao L., Li J., Song H. (2020). Deep-learning-enabled security issues in the internet of things. IEEE Internet Things J..

[69] Jiang H., Wang M., Zhao P., Xiao Z., Dustdar S. (2021). A Utility-Aware General Framework with Quantifiable Privacy Preservation for Destination Prediction in LBSs. IEEE/ACM Trans. Netw..

[70] Liu H., Yuan H., Liu Q., Hou J., Zeng H., Kwong S. (2022). A Hybrid Compression Framework for Color Attributes of Static 3D Point Clouds. IEEE Trans. Circuits Syst. Video Technol..

[71] Liu H., Yuan H., Hou J., Hamzaoui R., Gao W. (2022). PUFA-GAN: A Frequency-Aware Generative Adversarial Network for 3D Point Cloud Upsampling. IEEE Trans. Image Process..

[72] Mi C., Huang S., Zhang Y., Zhang Z., Postolache O. (2022). Design and Implementation of 3-D Measurement Method for Container Handling Target. J. Mar. Sci. Eng..

[73] Bao N., Zhang T., Huang R., Biswal S., Su J., Wang Y., Cha Y. (2023). A Deep Transfer Learning Network for Structural Condition Identification with Limited Real-World Training Data. Struct. Control Health Monit..

[74] Lv Z., Song H. (2019). Mobile internet of things under data physical fusion technology. IEEE Internet Things J..

[75] Lu S., Liu M., Yin L., Yin Z., Liu X., Zheng W., Kong X. (2023). The multi-modal fusion in visual question answering: A review of attention mechanisms. PeerJ Comput. Sci..

[76] Cheng B., Zhu D., Zhao S., Chen J. (2016). Situation-Aware IoT Service Coordination Using the Event-Driven SOA Paradigm. IEEE Trans. Netw. Serv. Manag..

